# Host-specific *Cryptosporidium*, *Giardia* and *Enterocytozoon bieneusi* in shelter dogs from central Europe

**DOI:** 10.1017/S003118202400009X

**Published:** 2024-04

**Authors:** Magdalena Szydłowicz, Żaneta Zajączkowska, Antonina Lewicka, Błażej Łukianowski, Mateusz Kamiński, Nikola Holubová, Bohumil Sak, Martin Kváč, Marta Kicia

**Affiliations:** 1 Department of Biology and Medical Parasitology, Wroclaw Medical University, Wroclaw, Poland; 2 Department of Pathomorphology, 4th Military Clinical Hospital, Wroclaw, Poland; 3 Biology Centre, Academy of Sciences of the Czech Republic, České Budějovice, Czech Republic; 4 Faculty of Agriculture and Technology, University of South Bohemia in České Budějovice, České Budějovice, Czech Republic

**Keywords:** 60-kDa glycoprotein, glutamate dehydrogenase, internal transcribed spacer region of rRNA, intestinal protists, opportunistic pathogens, PCR, small ribosomal subunit rRNA, subtyping, triosephosphate isomerase, *β*-giardin

## Abstract

*Cryptosporidium* spp., *Giardia intestinalis* and microsporidia are unicellular opportunistic pathogens that can cause gastrointestinal infections in both animals and humans. Since companion animals may serve as a source of infection, the aim of the present screening study was to analyse the prevalence of these intestinal protists in fecal samples collected from dogs living in 10 animal shelters in central Europe (101 dogs from Poland and 86 from the Czech Republic), combined with molecular subtyping of the detected organisms in order to assess their genetic diversity. Genus-specific polymerase chain reactions were performed to detect DNA of the tested species and to conduct molecular subtyping in collected samples, followed by statistical evaluation of the data obtained (using *χ*^2^ or Fisher's tests). The observed prevalence was 15.5, 10.2, 1 and 1% for *G. intestinalis*, *Enterocytozoon bieneusi*, *Cryptosporidium* spp. and *Encephalitozoon cuniculi*, respectively. Molecular evaluation has revealed the predominance of dog-specific genotypes (*Cryptosporidium canis* XXe1 subtype; *G. intestinalis* assemblages C and D; *E. cuniculi* genotype II; *E. bieneusi* genotypes D and PtEbIX), suggesting that shelter dogs do not pose a high risk of human transmission. Interestingly, the percentage distribution of the detected pathogens differed between both countries and individual shelters, suggesting that the risk of infection may be associated with conditions typical of a given location.

## Graphical abstract

**Figure:**
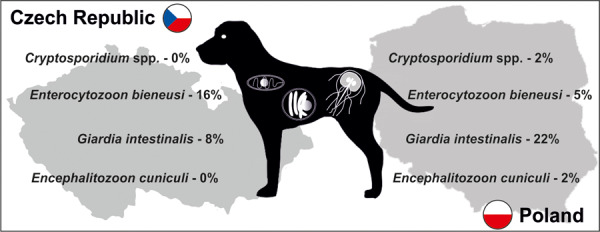

## Introduction

Dogs play an important role in human life as companion animals; however, they may be carriers of various pathogens, constituting a potential reservoir of zoonotic infections for their owners. Many of these infectious agents reside in the intestinal tract; therefore, their dispersive forms are excreted with animal stool and may easily be spread to other hosts by fecal–oral transmission through direct contact or indirectly *via* water or food contamination. This includes unicellular enteric organisms such as *Giardia intestinalis* (syn. *duodenalis* or *lamblia*), *Cryptosporidium* spp. or microsporidia from genera *Encephalitozoon* and *Enterocytozoon*, which can be responsible for gastrointestinal symptoms like abdominal pain, diarrhoea or flatulence (Xiao, 2010; Liao *et al*., 2020). Since these organisms belong to the group of opportunistic pathogens, infection is of particular importance for individuals with impaired immunity (e.g. HIV-infected patients, cancer-treated patients or transplant recipients), in whom it may lead to the development of hazardous, even life-threatening symptoms.

*Giardia intestinalis* is the most common intestinal pathogenic protozoan in humans and animals (Kváč *et al*., 2017), consisting of 8 distinct assemblages (A–H) differing in host specificities. Assemblages A and B display a broad host range and are most commonly reported in humans, while the remaining 6 seem to be host-specific for non-human species, with assemblages C and D predominantly found in dogs (Bouzid *et al*., 2015; Ryan and Zahedi, 2019). Among nearly 51 valid *Cryptosporidium* species (Tůmová *et al*., 2023), *Cryptosporidium hominis* and *Cryptosporidium parvum* represent the major causes of human cryptosporidiosis, whereas *Cryptosporidium meleagridis*, *Cryptosporidium mortiferum*, *Cryptosporidium felis* and *Cryptosporidium canis* are rare causative agents of zoonotic infections, with the latter being the most prevalent in dogs (Xu *et al*., 2016; Li *et al*., 2021; Alderisio *et al*., 2023). Out of over 1200 microsporidian species described so far, *Enterocytozoon bieneusi* and *Encephalitozoon* genus, including *Encephalitozoon intestinalis*, *Encephalitozoon cuniculi* and *Encephalitozoon hellem*, represent the species causing human microsporidiosis (Didier *et al*., 2000), especially in persons with impaired immunity (Kicia *et al*., 2014, 2016). These species may also be detected in a broad range of other hosts (livestock, wildlife and domesticated animals) (Dengjel *et al*., 2001; Mathis *et al*., 2005). The phylogenetic analysis of *E. bieneusi* allows for distinction of various genotypes differing in a host specificity. Genotypes D, EbpC and type IV are characterized by the widest host range and are also most frequently reported in humans (Li *et al*., 2019). In turn, genotype PtEbIX seems to be restricted to canine host population (Li *et al*., 2019), although dogs can serve as a reservoir of many other zoonotic genotypes as well, including those most often reported in humans (Li *et al*., 2020). Regarding the genus *Encephalitozoon*, the ability of these 3 species to inhabit a wide variety of organisms has been shown, with *E. cuniculi* having the widest host range, mainly among mammals and birds. Four *E. cuniculi* strains have been identified (I–IV); ‘canine’ strain III has been shown to cause high mortality in dogs, while the recently discovered ‘human’ strain IV has so far been documented in humans, cats and dogs. Although there appears to be some host preference in each strain, this specificity is not exact; humans have been found to be infected with all known strains (though rarely with strain III). In turn, *E. hellem* is the most common species among birds, while *E. intestinalis* is the most prevalent *Encephalitozoon* species in humans (Hinney *et al*., 2016).

The dispersive forms of the discussed pathogens are very resistant, and many species of wild and domesticated animals, as well as humans, may serve as their hosts which facilitates their spread and maintenance in the environment. Previous research on the occurrence of these species in central Europe, including Poland and the Czech Republic, shows different data depending on the population studied and the detection methods used. According to a review by Plutzer *et al*., the reported incidence of *Cryptosporidium* spp. and *G. intestinalis* per 100 000 inhabitants is 0.006 and 5.43 for Poland and 0.01 and 0.51 for the Czech Republic, respectively (Plutzer *et al*., 2018). In Poland, estimates of the prevalence of these 2 species in humans are available based on the results of research limited to specific population groups and regions (Plutzer *et al*., 2018). However, studies of Czech residents regarding the seroprevalence of *Cryptosporidium* spp. show the frequency of antibodies at the level of approximately 67–72% (Kozisek *et al*., 2008). In turn, the prevalence of *Cryptosporidium* spp. in dogs in Poland ranges from approximately 3.5 to 12.5% (Bajer and Bednarska, 2007; Piekara-Stępińska *et al*., 2021*a*), and *G. intestinalis* from 6 to 36%, with mainly canid-specific genotypes detected, which suggests that they do not represent an important source of *Giardia* infection for humans (Bajer and Bednarska, 2007; Piekarska *et al*., 2016; Piekara-Stępińska *et al*., 2021*b*). A similar prevalence refers to dogs from the Czech Republic, especially those from animal shelters (Zemanová *et al*., 2005; Dubná *et al*., 2007). Microsporidia, however, occur in dogs at a low level, only a few per cent, which may nevertheless pose a risk for immunodeficient individuals, as zoonotic genotypes are often detected (Piekarska *et al*., 2017). Importantly, frequent exposure to microsporidia has been confirmed among immunocompetent people in the Czech Republic (Sak *et al*., 2011), while in studies conducted in Poland, up to 26% of tested immunocompromised individuals were found to be infected with at least 1 microsporidian species (Kicia *et al*., 2016, 2019).

It has been previously shown that specific genotypes and assemblages of these enteric pathogens may be detected in both humans and animals (Xiao *et al*., 2007; Soliman *et al*., 2011; Karim *et al*., 2014*a*, 2014*b*; Hinney *et al*., 2016), suggesting the possible zoonotic route of transmission. One of their sources may be shelter dogs, which often live in poor sanitary conditions and crowded spaces that favour the spread of such microorganisms (Raza *et al*., 2018). Currently, there are 226 registered animal shelters in Poland (General Veterinary Inspectorate, 2023) and 248 in the Czech Republic (State Veterinary Administration, 2024). Therefore, the aim of the present study was to investigate the occurrence of *Cryptosporidium* spp., *G. intestinalis*, *Encephalitozoon* spp. and *E. bieneusi* in dogs living in animal shelters in central Europe (Poland and the Czech Republic) and to assess the host specificity and zoonotic potential of these organisms at the genotype level.

## Materials and methods

### Samples

Individual fresh fecal samples were collected from dogs in animal shelters in Poland and the Czech Republic. Samples were collected directly from the floor by study research staff immediately after defecation, with care taken to avoid sampling fecal material that came into contact with the ground (concrete surface, without contact with the soil). They were collected in the morning, before daily cleaning routinely performed in each shelter. Each sample was individually placed in a sterile tube with animal ID, refrigerated at 4°C without preservatives and transported to laboratory. None of the collected stool had an apparent diarrhoeal symptom at the time of sampling. Where possible, information about the animal, such as sex and age, was also recorded during material collection (see Supplementary Table 1). Control of intestinal protozoa in dogs and cats in both Polish and Czech shelters is carried out according to current ESCCAP guidelines (ESCCAP, 2018) – in all facilities, pyrantel and fenbendazole were routinely used once every 3 months as part of antiparasitic prophylaxis.

### Molecular analysis

Stool samples were stored up to 2 months in 4°C without preservatives until DNA extraction. Initial homogenization of 200 mg of each stool sample was performed by bead disruption for 60 s at 5.5 m s^−1^ with 0.5 mm glass beads using a Precellys 24 Instrument (Bertin Technologies, Montigny le Bretonneux, France), followed by genomic DNA (gDNA) extraction using a GeneMATRIX Stool DNA Purification Kit (EurX, Gdańsk, Poland). Molecular detection was based on the nested polymerase chain reaction (PCR) protocols for the amplification of the chosen genes of *E. bieneusi* (*ITS*), *Encephalitozoon* spp. (*ITS*), *Cryptosporidium* spp. (18S rRNA) and *G. intestinalis* (*TPI*) (Didier *et al*., 1995; Katzwinkel-Wladarsch *et al*., 1996; Xiao *et al*., 1999; Buckholt *et al*., 2002; Sulaiman *et al*., 2003). Additional PCRs amplifying selected loci were performed for subtyping in order to assess intra-species genetic diversity in the case of samples positive for *Cryptosporidium* spp. (partial 60-kDa glycoprotein gene – *gp60*) and *G. intestinalis* (*β*-giardin – *BG* and glutamate dehydrogenase – *GDH*) (see Supplementary Table 2) (Cacciò *et al*., 2002, 2008; Lalle *et al*., 2005; Jiang *et al*., 2021). Each PCR contained 0.25–2.0 μL of DNA, 200 μm each of deoxynucleoside triphosphate (dNTP), 1× PCR buffer (DreamTaq™ Green Buffer, ThermoFisher Scientific, Waltham, MA, USA), 3.0 mm MgCl_2_, 0.125 U of Taq polymerase (ThermoFisher Scientific), 10 μg of bovine serum albumin (BSA) and 200 nm of each primer in a total of 20–25 μL reaction. The reactions were performed in a C1000 Bio-Rad thermocycler, with an initial hot start (94°C for 5 min) and a final extension (72°C for 10 min), according to the conditions described in Supplementary Table 2. An aliquot of primary PCR was used as a template for the secondary PCR. Its conditions were identical to the primary PCR, except that BSA was not added to the secondary reaction. Negative (molecular grade water) and positive controls (DNA extracted from *E. bieneusi* genotype CZ3, *E. cuniculi* genotype III spores, *Cryptosporidium serpentis* oocysts or *G. intestinalis* assemblage F cysts) were included in each PCR amplification. Secondary PCR products were electrophoresed on a 1% agarose gel containing 0.2 mg mL^−1^ Midori Green DNA stain in TAE buffer at 75 V for approximately 1 h. Bands of the predicted sizes were visualized using a UV light source, cut from the gel, extracted using a Zymoclean Gel DNA Recovery Kit (Zymo Research, Irvine, CA, USA) and sequenced bi-directionally by a company offering this service commercially (Genomed S.A., Warsaw, Poland). The nucleotide sequences obtained were processed using Chromas Pro 2.4.1 software (Technelysium, Pty, Ltd., South Brisbane, Australia). Subsequently, BLAST analysis (https://blast.ncbi.nlm.nih.gov/Blast.cgi) was performed to verify the identity of the sequences. The edited and aligned sequences were further processed using BioEdit v.7.0.5 (Hall, 1999). To align the obtained sequences with reference sequences from GenBank, the online server MAFFT version 7 was used (http://mafft.cbrc.jp/alignment/software). The best model for DNA/protein phylogeny for each alignment was selected based on the Bayesian information criterion in MEGA 7 (Kumar *et al*., 2016). Tamura's 3-parameter model + G + I was used for the alignments. The maximum likelihood (ML) approach was carried out in MEGA7 software. Bootstrap support was calculated based on 1000 replications to evaluate the robustness of tree branching. Finally, the resulting trees were visualized using Corel Draw X7 software (https://www.corel.draw.com). Representative nucleotide sequences of all loci used as markers for subtyping of isolates obtained in the current study were deposited in GenBank with the accession numbers OR791083, OR791084, OR791770, OR791659, OR791771–OR791785, OR807726 and OR807727.

### Statistical analysis

Statistical analysis was performed using *χ*^2^ or Fisher's tests to compare the frequency of occurrence of the tested pathogens between Polish and Czech shelters (Statistica software, TIBCO Software Inc., USA). A *P* < 0.05 was considered significant.

## Results

**Figure 1.**
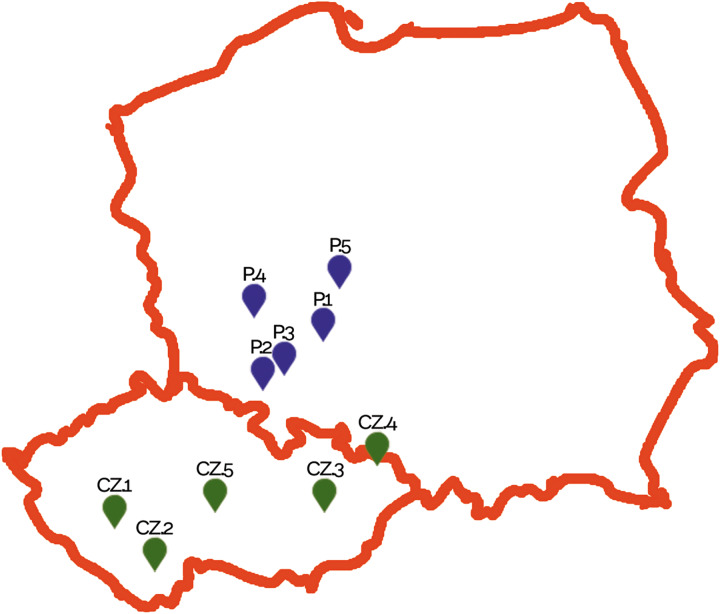
Schematic arrangement of shelters in Poland (P.1–P.5) and the Czech Republic (CZ.1–CZ.5) from which samples were collected.

A total of 187 apparently healthy dogs from 10 shelters (Fig. 1), 5 in Poland (101 dogs) and 5 in the Czech Republic (86 dogs), were studied (Table 1). Specific DNA of targeted parasites was detected in 50 of 187 animals (26.7%), with higher occurrence observed in Polish than in Czech animals (32.6 *vs* 24.4%, *χ*^2^ = 1.0254; *P* *=* 0.3112; Table 1). Most of the detected infections were monoinfections; only 2 dogs (ID 2966_CZ.1 and 3031_CZ.5) had a coinfection, *E. bieneusi* and *G. intestinalis. Giardia intestinalis* (29 dogs, 15.5%) followed by *E. bieneusi* (19 dogs, 10.2%) were the most frequently detected parasites, whereas each *Encephalitozoon* spp. and *Cryptosporidium* spp. were found in 2 dogs (Table 1). Overall, there was a significant trend towards more frequent occurrence of *G. intestinalis* in Polish (21.8%) *vs* Czech animals (8.1%, *χ*^2^ = 6.5979; *P* *=* 0.0102), while *E. bieneusi* was seen more often in dogs from Czech shelters (16.3%) than in Polish ones (4.9%, *χ*^2^ = 6.5305; *P* = 0.0106). Since detailed demographic data were obtained only from a small number of individuals, they were not subjected to statistical analysis.

**Table 1.** Occurrence of *Cryptosporidium* spp., *Giardia intestinalis*, *Encephalitozoon cuniculi* and *Enterocytozoon bieneusi* in individual dogs kept in animal shelters in Poland and the Czech Republic

Country	Location	No. of tested animals	Dogs ID	No. of dogs positive for screened parasites			
				*Cryptosporidium* spp.	*G. intestinalis*	*E. cuniculi*	*E. bieneusi*
Poland	P.1	39	2806	1	–	–	–
			2803, 2807	–	2	–	–
			2839	–	–	1	–
			2808, 2834				2
	P.2	32	2728, 2732, 2733, 2742, 2744, 2747, 2748, 2751, 2753, 2757	–	10	–	–
			2735	1	–	–	–
			2727	–	–	–	1
	P.3	13	2768, 2771, 2772, 2773, 2775, 2778, 2779, 2780	–	8	–	–
	P.4	11	2783	–	–	1	–
			2787	–	1	–	–
			2789	–	–	–	1
	P.5	6	2696	–	1	–	–
			2699	–	–	–	1
Czech Republic	CZ.1	20	2966	–	1	–	1
			2965, 2973, 2977	–	–	–	3
	CZ.2	7	2985, 2986, 2987, 2989	–	–	–	4
	CZ.3	6	–	–	–	–	–
	CZ.4	30	3010, 3027	–	2	–	–
	CZ.5	23	3031	–	1	–	1
			3030, 3036, 3041	–	3	–	–
			3032, 3044, 3047, 3048, 3049	–	–	–	5
Total		187		2 (1.1%)	29 (15.5%)	2 (1.1%)	19 (10.2%)

**Figure 2.**
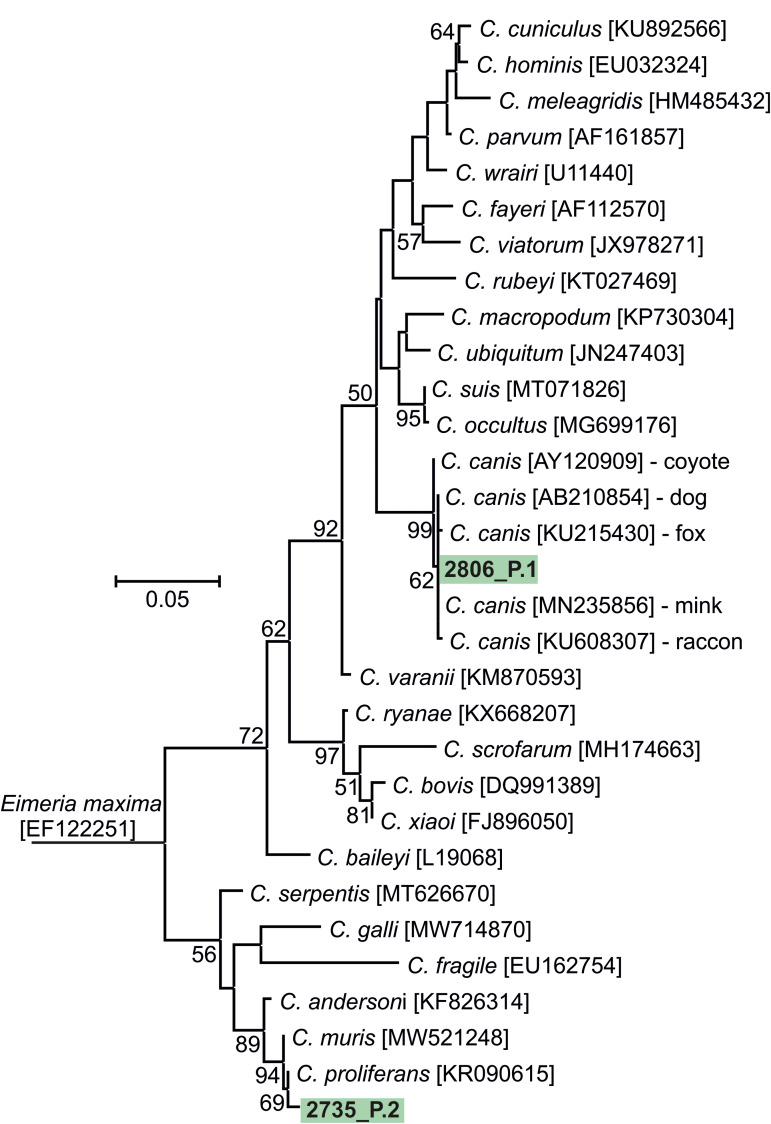
Phylogenetic relationships between *Cryptosporidium* spp. detected in dogs in this study (highlighted in green) and other *Cryptosporidium* available in GenBank using an ML analysis of partial sequences of 18S rRNA (sequence alignment length: 820 bp). Percentage supports (>50%) from 1000 pseudoreplicates are indicated next to the supported node. The branch length scale bar indicates the number of substitutions per site. Sequences from this study are identified by an isolate number (e.g. 2806) followed by region and location (P.1, Poland location 1).

**Figure 3.**
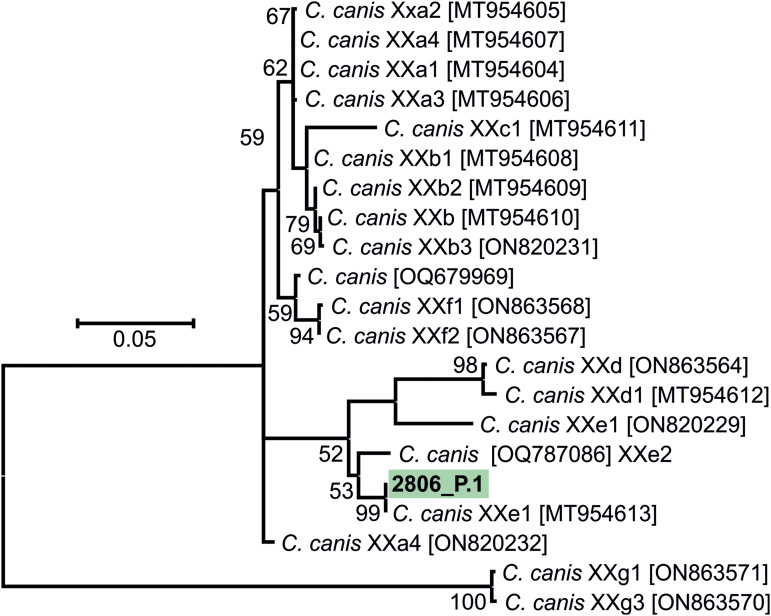
Phylogenetic relationship between *Cryptosporidium canis* detected in 1 dog in this study (highlighted in green) and other *C. canis* available in GenBank using an ML analysis of a region of *gp60* gene (sequence alignment length: 540 bp). Percentage supports (>50%) from 1000 pseudoreplicates are indicated next to the supported node. The branch length scale bar indicates the number of substitutions per site. Sequences from this study are identified by an isolate number (2806) followed by region and location (P.1, Poland location 1).

**Figure 4.**
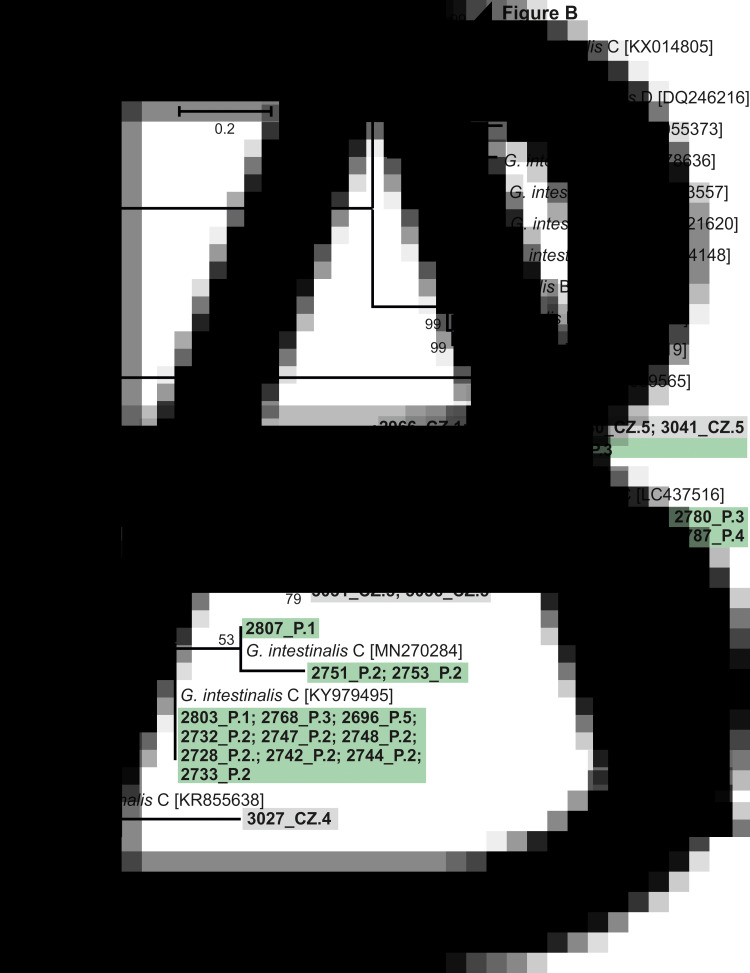
Phylogenetic relationships between *Giardia intestinalis* assemblages detected in dogs in this study (highlighted in green – Poland or in grey – Czech Republic) and other *G. intestinalis* assemblages available in GenBank using an ML analysis of a region of *TPI* gene (sequence alignment length: 467 bp). Percentage supports (>50%) from 1000 pseudoreplicates are indicated next to the supported node. The branch length scale bar indicates the number of substitutions per site. Sequences from this study are identified by an isolate number (e.g. 2966) followed by region and location (P.1, Poland location 1, CZ.1, Czech Republic location 1).

**Figure 5.**
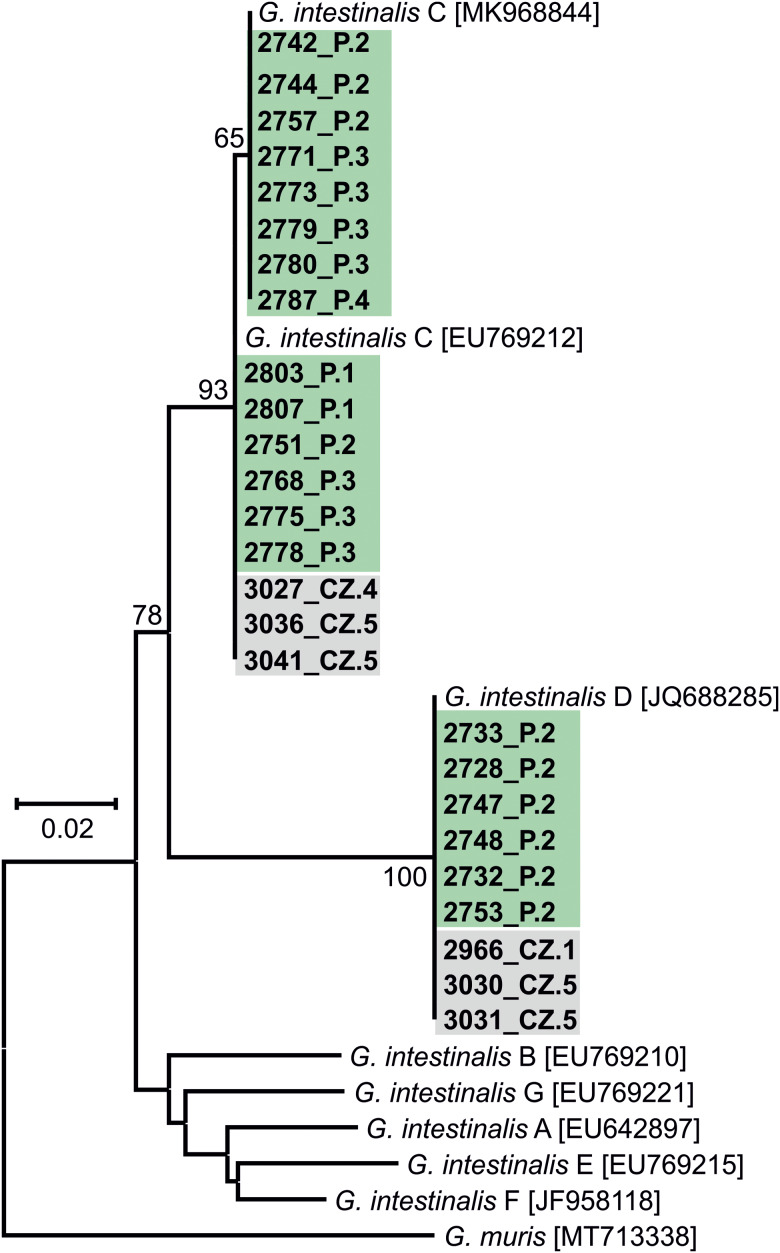
Phylogenetic relationships between *G. intestinalis* assemblages detected in dogs in this study (highlighted in green – Poland or in grey – Czech Republic) and other *G. intestinalis* assemblages available in GenBank using an ML analysis of a region of *BG* gene (sequence alignment length: 820 bp). Percentage supports (>50%) from 1000 pseudoreplicates are indicated next to the supported node. The branch length scale bar indicates the number of substitutions per site. Sequences from this study are identified by an isolate number (e.g. 2966) followed by region and location (P.1, Poland location 1, CZ.1, Czech Republic location 1).

**Figure 6.**
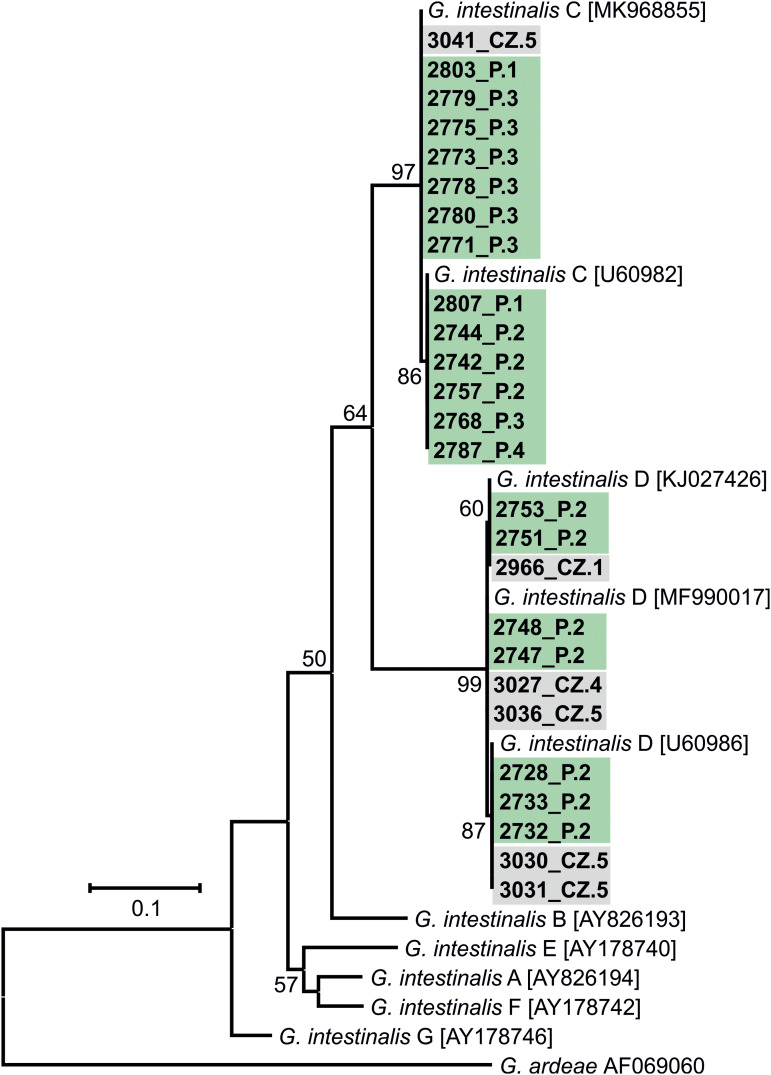
Phylogenetic relationships between *G. intestinalis* assemblages detected in dogs in this study (highlighted in green – Poland or in grey – Czech Republic) and other *G. intestinalis* assemblages available in GenBank using an ML analysis of a region of *GDH* gene (sequence alignment length: 439 bp). Percentage supports (>50%) from 1000 pseudoreplicates are indicated next to the supported node. The branch length scale bar indicates the number of substitutions per site. Sequences from this study are identified by an isolate number (e.g. 2966) followed by region and location (P.1, Poland location 1, CZ.1, Czech Republic location 1).

**Figure 7.**
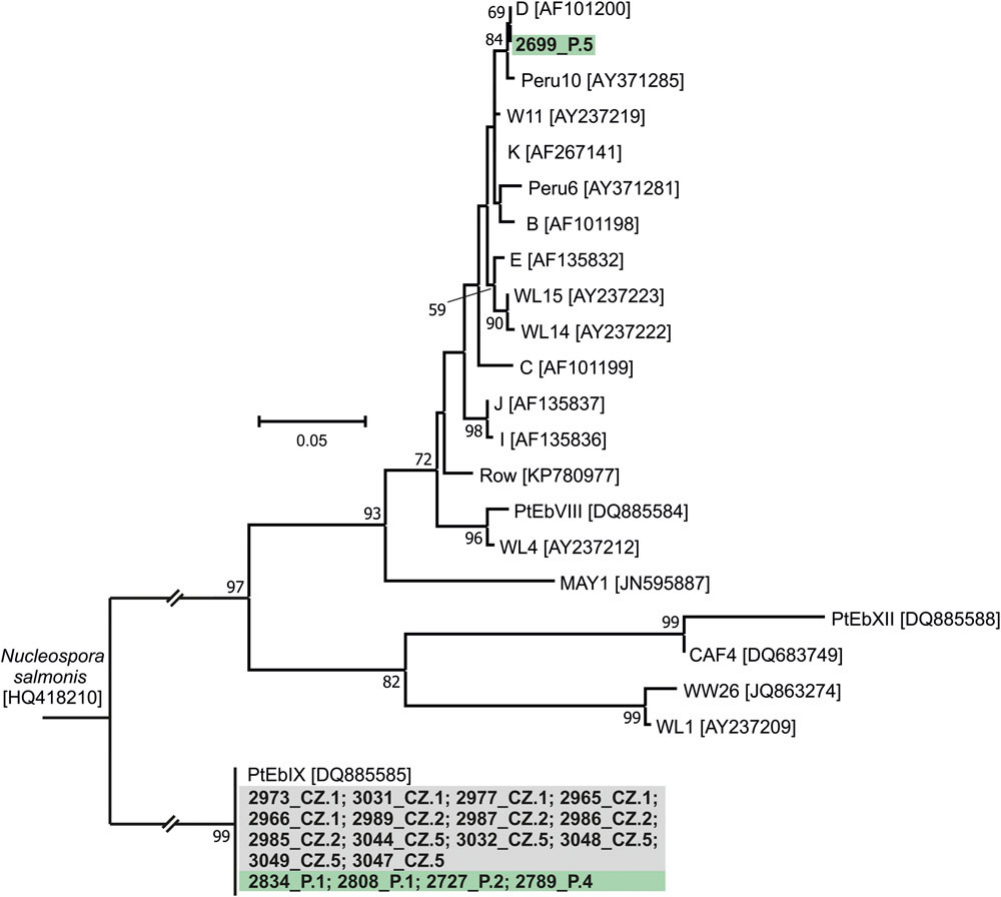
Phylogenetic relationships between *Enterocytozoon bieneusi* genotypes detected in dogs in this study (highlighted in green – Poland or in grey – Czech Republic) and other *E. bieneusi* genotypes available in GenBank using an ML analysis of *ITS* region of rRNA gene (sequence alignment length: 309 bp). Percentage supports (>50%) from 1000 pseudoreplicates are indicated next to the supported node. The branch length scale bar indicates the number of substitutions per site. Sequences from this study are identified by an isolate number (e.g. 2973) followed by region and location (P.1, Poland location 1, CZ.1, Czech Republic location 1).

The results of genotyping of all pathogens performed in this study are presented in Table 2. Phylogeny analysis of partial sequences of 18S rRNA of *Cryptosporidium* showed the presence of *C. canis* identical to *C. canis* dog genotype (GenBank Acc. No. AB120909) in isolate 2806_P.1 and in a *Cryptosporidium* sp. isolate 2735_P.2, phylogenetically clustered near the gastric *Cryptosporidium* spp. (Fig. 2). *Cryptosporidium* sp. isolate 2735_P.2 differed from *Cryptosporidium proliferans* in 3 single-nucleotide polymorphisms (SNPs) with 99.6% sequence identity. Based on *C. canis gp60* locus subtyping, isolate 2806_P.1 was assigned to the XXe family (Fig. 3). Genotype II, determined at *ITS* sequences, was detected in both dogs positive for *E. cuniculi* (Table 2; phylogeny is not shown). Phylogenetic analysis of *Giardia* showed different results in 12 isolates, depending on the marker used (Table 2). Subtyping based on *TPI* gene revealed the presence of only assemblage C in all samples examined (Fig. 4), while for *BG* (Fig. 5) and *GDH* (Fig. 6) loci the presence of both C and D assemblages has been shown. Subtyping of *BG* and *GDH* failed in 2 and 3 isolates, respectively. With the exception of genotype D, which was detected in dog 2699_P.5, all other *E. bieneusi* sequences were identical to the PtEbIX genotype (Fig. 7).

**Table 2.** Results of genotyping of *Cryptosporidium* spp., *G. intestinalis*, *E. cuniculi* and *E. bieneusi* in all tested samples

		*G. intestinalis* assemblage			*Cryptosporidium* spp. species/subtype		*E. cuniculi* genotype *ITS*	*E. bieneusi* genotype *ITS*
Country	Dog ID_ Location	*TPI*	*BG*	*GDH*	18S rRNA	*gp60*		
Poland	2803_P.1	C [OR791776]	C [OR791779]	C				
	2806_P.1				*Cryptosporidium canis* [OR791083]	XXe1 [OR791770]		
	2807_P.1	C [OR791774]	C	C [OR791782]				
	2808_P.1							PtEbIX
	2834_P.1							PtEbIX [OR807727]
	2839_P.1						II [OR791659]	
	2728_P.2	C	D	D [OR791785]				
	2732_P.2	C	D	D				
	2733_P.2	C	D [OR791780]	D				
	2735_P.2				*Cryptosporidium proliferans*^a^ [OR791084]			
	2742_P.2	C	C [OR791778]	C				
	2744_P.2	C	C	C				
	2747_P.2	C	D	D				
	2748_P.2	C	D	D [OR791784]				
	2751_P.2	C [OR791775]	C	D				
	2753_P.2	C	D	D [OR791783]				
	2757_P.2	C	C	C				
	2768_P.3	C	C	C				
	2771_P.3	C	C	C				
	2772_P.3	C	N/A^b^	N/A^b^				
	2773_P.3	C	C	C				
	2775_P.3	C	C	C				
	2778_P.3	C [OR791772]	C	C				
	2779_P.3	C	C	C				
	2780_P.3	C	C	C				
	2783_P.4						II	
	2787_P.4	C	C	C				
	2789_P.4							PtEbIX
	2696_P.5	C	N/A^b^	N/A^b^				
	2699_P.5							D [OR807726]
Czech Republic	2965_CZ.1							PtEbIX
	2966_CZ.1	C [OR791771]	D	D				PtEbIX
	2973_CZ.1							PtEbIX
	2977_CZ.1							PtEbIX
	2985_CZ.2							PtEbIX
	2986_CZ.2							PtEbIX
	2987_CZ.2							PtEbIX
	2989_CZ.2							PtEbIX
	3010_CZ.4	C	D	N/A^b^				
	3027_CZ.4	C [OR791777]	C	D				
	3030_CZ.5	C	D	D				
	3031_CZ.5	C	D	D				PtEbIX
	3032_CZ.5							PtEbIX
	3036_CZ.5	C [OR791773]	C	D				
	3041_CZ.5	C	C	C [OR791781]				
	3044_CZ.5							PtEbIX
	3047_CZ.5							PtEbIX
	3048_CZ.5							PtEbIX
	3049_CZ.5							PtEbIX

Accession numbers in square brackets indicate the isolates deposited in GenBank as representative nucleotide sequences derived from the present study.

*TPI*, triosephosphate isomerase; *BG*, *β*-giardin; *GDH*, glutamate dehydrogenase; 18S rRNA, small ribosomal subunit rRNA; *gp60*, 60-kDa glycoprotein; *ITS*, internal transcribed spacer region of rRNA.

a *Cryptosporidium* sp. isolate 2735_P.2, phylogenetically clustered near the gastric *Cryptosporidium* spp., differed from *C. proliferans* in 3 SNPs with 99.6% sequence identity of the 18S rRNA region.

b N/A, assemblage not available (subtyping failed).

## Discussion

In the present study, the screening of fecal samples collected from shelter dogs in central Europe was performed in order to analyse the prevalence of zoonotic unicellular pathogens (*Cryptosporidium* spp., *G. intestinalis*, *E. bieneusi* and *Encephalitozoon* spp.) at the molecular level. Over a quarter of the tested dogs were carriers of at least one of the studied pathogens, among which the most often observed was *G. intestinalis* – one of the most common intestinal parasites infecting humans and animals (Kváč *et al*., 2017). Nevertheless, its prevalence in other European canine populations, including shelter dogs, has shown to be even higher [27–36.5% in various regions of Spain (Gil *et al*., 2017; Adell-Aledón *et al*., 2018; Remesar *et al*., 2022), 33.8% in Portugal (Pereira *et al*., 2021), over 45% in Serbia (Sommer *et al*., 2017), while in central Italy this value ranged from about 7% (Scaramozzino *et al*., 2018) to 41% (Agresti *et al*., 2022)]. Generally, such a high prevalence is most likely due to the simple and direct life cycle of *Giardia*, with easily spread dispersive forms excreted in feces, which facilitates transmission in highly dense populations, such as those found in animal shelters. *Enterocytozoon bieneusi* was also found to be a common pathogen in dogs in the present study, with an overall prevalence of 10.2%, which agrees with previous reports considering European dogs, with infection rates ranging between 4.9 and 11.7% (Mathis *et al*., 2005; Santín and Fayer, 2011; Piekarska *et al*., 2017). In turn, considerably low prevalences were observed for *Encephalitozoon* ssp. and *Cryptosporidium* spp. (~1% for both), comparably to previous reports regarding canine populations: 0–2.4% for *Encephalitozoon* spp. (Piekarska *et al*., 2017; Delrobaei *et al*., 2019) and 0.6–4.9% for *Cryptosporidium* spp. (Giangaspero *et al*., 2006; Simonato *et al*., 2017; Yu *et al*., 2018; Piekara-Stępińska *et al*., 2021*a*), although in 1 German study the *Cryptosporidium* prevalence was as high as 10% (Murnik *et al*., 2022). Notwithstanding, the true prevalence of these pathogens among healthy hosts may, in fact, be higher as their forms are excreted periodically and irregularly, which may be overlooked with a single sampling (Sak *et al*., 2010). It would therefore be recommended to collect samples several times from the same animals, which may prove difficult due to the conditions specific to the shelters, such as the rotation of animals or irregular hours of cleaning the excrement. Nevertheless, the fact that the demonstrated prevalence of pathogens such as *G. intestinalis* was high even with only 1 sampling underscores the importance of their likely distribution in the population and the wide reservoir of the pathogen. Differences in the frequencies of the studied species between Polish and Czech dogs, as well as the higher prevalence of specific pathogens in individual facilities, may be related to some specific conditions typical of a particular location.

Sequence analyses of detected pathogens showed that most infections involved dog-specific genotypes or species, the transmission of which may be favoured by intensive contact among large numbers of dogs living together. In the case of 12 isolates, assignment to the appropriate *G. intestinalis* assemblage was difficult because subtyping results varied depending on the locus used. Similar discrepancies have been reported in previous studies (Read *et al*., 2004; Traub *et al*., 2004), emphasizing the importance of using multilocus genotyping in the molecular analysis of *Giardia* diversity. Nevertheless, all *G. intestinalis*-positive samples harboured assemblage C or D, which have a strong host specificity for dogs and other canines (Feng and Xiao, 2011). These assemblages were also found to be highly prevalent in different dog populations in Europe (Simonato *et al*., 2015; Adell-Aledón *et al*., 2018; Pereira *et al*., 2021) and although sporadically they have been reported in humans as well (Broglia *et al*., 2013; Liu *et al*., 2014; Villamizar *et al*., 2019), their zoonotic relevance seems to be low and limited to individuals at risk, for instance, children or immunocompromised persons. Likewise, cases of *C. canis* colonization in humans were described in individuals at increased risk (children, HIV-infected adults) and immunocompetent people as well (Learmonth *et al*., 2004; Gatei *et al*., 2007; Feng *et al*., 2012; Liao *et al*., 2020). However, due to the relatively transient nature of these infections in humans, dogs do not seem to represent an important source of cryptosporidiosis for people (Villamizar *et al*., 2019; Liao *et al*., 2020). To date, 9 families of *C. canis* subtypes (XXa–XXi) have been identified based on *gp60* locus subtyping, occurring not only in canids, but also in minks, foxes and humans (Jiang *et al*., 2021; Murnik *et al*., 2022; Wang *et al*., 2022). According to the study of Jiang *et al*., the zoonotic potential may concern XXa family, detected in both dog and human samples (Jiang *et al*., 2021). In our study, analysis of the *C. canis gp60* locus in the 2806_P.1 isolate revealed that it belongs to the XXe family (Fig. 3), which was also the most prevalent among dogs in the report from Germany (Murnik *et al*., 2022). In turn, the newly detected *Cryptosporidium* sp. isolate 2735_P.2 also does not pose a significant risk to humans and probably not to dogs as well. An incidental infection/contamination was likely caused by rodent feces. *Cryptosporidium* sp. isolate 2735_P.2 is closely related to *Cryptosporidium muris* and *C. proliferans*, whose hosts are rodents. However, the phylogenetic position is not related to host specificity, and therefore another host cannot be excluded (Kváč *et al*., 2018). To summarize the subtyping results, as in previous studies (de Lucio *et al*., 2017; Rehbein *et al*., 2019), zoonotic transmission of giardiasis or cryptosporidiosis between dogs and humans is most likely a rare event.

All *E. bieneusi* isolates detected in the studied dogs, except for 1 clustering to genotype D, were identical to *E. bieneusi* genotype PtEbIX. This genotype appears to be specific to dogs; to date, it has been detected almost exclusively in dogs and sporadically in wolves, cats and swans (Santín *et al*., 2008; Abe *et al*., 2009; Santín and Fayer, 2011; Mori *et al*., 2013; Karim *et al*., 2014*a*; Piekarska *et al*., 2017; Kváč *et al*., 2021). In turn, both *E. bieneusi* genotype D and *E. cuniculi* genotype II detected in the present study have been reported in a broad range of hosts so far, including humans (Li *et al*., 2012; Kváč *et al*., 2017; Piekarska *et al*., 2017; Delrobaei *et al*., 2019). Observation of microsporidian genotypes with a human-infection capacity in companion animals suggests that pets may be of importance as one of the potential sources of infection. However, the presented results do not indicate that dogs in shelters in Poland and the Czech Republic represent a significant source of zoonotic species and genotypes of the studied parasites for humans.

Our study had some limitations. Firstly, due to its screening nature, a detailed analysis in the context of the demographic data of the tested dogs or the drugs used was not possible to conduct. Moreover, the study groups differed in size – in Poland it was possible to collect material from a larger number of animals than in the Czech Republic, which may have an impact on the differences in prevalence.

This study included clinically healthy animals without signs of intestinal infection, yet dispersive forms of potentially pathogenic and infectious organisms were observed. It should also be borne in mind that asymptomatic hosts could shed cysts, oocysts or spores occasionally and irregularly, and thus their screening with multiple sampling could increase the real observed prevalence (Sak *et al*., 2010). Nevertheless, since infections of the studied pathogens in dogs can often be asymptomatic, they may not be detected by routine veterinary examinations, and their occurrence in companion animals may be underestimated. On the contrary, the majority of species and genotypes observed in canine samples herein are not commonly associated with human infections, and aforesaid transmission routes seem to be rare. The exceptions are genotypes D (*E. bieneusi*) and II (*E. cuniculi*) observed in the present study, whose zoonotic potential should be emphasized due to their occurrence in a wide range of different hosts (Hinney *et al*., 2016; Li *et al*., 2019, 2020). Despite the low likelihood of transmission of the studied pathogens and due to the fact that they mainly affect immunosuppressed individuals, in whom the consequences of opportunistic infections may be life-threatening, the awareness among new dog owners is recommended, especially those with various levels of immunosuppression, on the relevance of diagnosing and treating zoonotic diseases.

## Supporting information

Szydłowicz et al. supplementary material 1 Szydłowicz et al. supplementary material

Szydłowicz et al. supplementary material 2 Szydłowicz et al. supplementary material

## Data Availability

The data that support the findings of this study are available from the corresponding author, MS, upon reasonable request.

## Supplementary material

The supplementary material for this article can be found at https://doi.org/10.1017/S003118202400009X
