# Supplementary material for: Host-specific Cryptosporidium, Giardia and Enterocytozoon bieneusi in shelter dogs from central Europe
Source: Parasitology. 2024 Feb 2;151(4):351–62. doi: 10.1017/S003118202400009X (PMC11044064; doi:10.1017/S003118202400009X)
Supplement: Szydłowicz et al. supplementary material 1 — Szydłowicz et al. supplementary material [file S003118202400009Xsup001.docx]

**Supplementary Table 1.** Standardized questionnaire regarding information about the investigated dogs, collected whenever possible.

| No. | Collection date | Dog ID | Sex | Age | Source shelter | Drugs used within last three months | Additional comments |
| --- | --- | --- | --- | --- | --- | --- | --- |
| 1. |  |  |  |  |  |  |  |
| 2. |  |  |  |  |  |  |  |
| 3. |  |  |  |  |  |  |  |
