# Supplementary material for: Host-specific Cryptosporidium, Giardia and Enterocytozoon bieneusi in shelter dogs from central Europe
Source: Parasitology. 2024 Feb 2;151(4):351–62. doi: 10.1017/S003118202400009X (PMC11044064; doi:10.1017/S003118202400009X)
Supplement: Szydłowicz et al. supplementary material 2 — Szydłowicz et al. supplementary material [file S003118202400009Xsup002.docx]

**Supplementary Table 2.** Oligonucleotide sequences and conditions used to amplify specific DNA of the tested pathogens.

| Species | Locus | Primer | | | Nested-PCR conditions | | | | | Reference |
| --- | --- | --- | --- | --- | --- | --- | --- | --- | --- | --- |
|  |  |  | Identity | Sequence (5’ 🡪 3’) | Denaturation | Annealing | Extension | Cycles (no.) | Amplicon  size (bp) |  |
| *Enterocytozoon bieneusi* | Internal transcribed spacer region (*ITS*) of rRNA^a^ | 1^o^ | **EBITS3** | GGTCATAGGGATGAAGAG | 94^o^C/45s | 57^o^C/30s | 72^o^C/60s | 35 | 435 | (Buckholt *et al.*, 2002) |
|  |  |  | **EBITS4** | TTCGAGTTCTTTCGCGCTC |  |  |  |  |  |  |
|  |  | 2^o^ | **EBITS1** | GCTCTGAATATCTATGGCT | 94^o^C/45s | 55^o^C/30s | 72^o^C/40s | 30 | 390 |  |
|  |  |  | **EBITS2.4** | ATCGCCGACGGATCCAAGTG |  |  |  |  |  |  |
| *Encephalitozoon* spp. | Internal transcribed spacer region  (*ITS*) of rRNA^a^ | 1^o^ | **INT580F** | TGCAGTTAAAATGTCCGTAGT | 94^o^C/45s | 55^o^C/30s | 72^o^C/60s | 35 | 1000 | (Didier *et al.*, 1995; Katzwinkel-Wladarsch *et al.*, 1996) |
|  |  |  | **INT580R** | TTTCACTCGCCGCTACTCAG |  |  |  |  |  |  |
|  |  | 2^o^ | **MSP3** | GGAATTCACACCGCCCGTCvyTAT | 94^o^C/45s | 55^o^C/30s | 72^o^C/40s | 35 | 289-315^b^ |  |
|  |  |  | **MSP4A** | CCAAGCTTATGCTTAAGTymAArGGGT |  |  |  |  |  |  |
| *Giardia intestinalis* | Triosephosphate isomerase (*TPI*)^a^ | 1^o^ | **AL3543** | AAATIATGCCTGCTGGTCG | 94^o^C/45s | 50^o^C/45s | 72^o^C/60s | 35 | 605 | (Sulaiman *et al.*, 2003) |
|  |  |  | **AL3546** | CAAACCTTITCCGCAAACC |  |  |  |  |  |  |
|  |  | 2^o^ | **AL3544** | CCCTTCATCGGIGGTAACTT | 94^o^C/45s | 50^o^C/45s | 72^o^C/60s | 35 | 530 |  |
|  |  |  | **AL3545** | GTGGCCACCACICCCGTGCC |  |  |  |  |  |  |
|  | Glutamate dehydrogenase (*GDH*) | 1^o^ | **GDH1**  **GDH2** | TTCCGTRTYCAGTACAACTC  ACCTCGTTCTGRGTGGCGCA | 94^o^C/45s | 55^o^C/30s | 72^o^C/60s | 35 | 754 | (Cacciò *et al.*, 2008) |
|  |  | 2^o^ | **GDH3**  **GDH4** | ATGACYGAGCTYCAGAGGCACGT  GTGGCGCARGGCATGATGCA | 94^o^C/45s | 55^o^C/30s | 72^o^C/60s | 35 | 532 |  |
|  | β-giardin (*BG*) | 1^o^ | **G7**  **G759** | AAGCCCGACGACCTCACCCGCAGTGC  GAGGCCGCCCTGGATCTTCGAGACGAC | 94^o^C/45s | 62^o^C/30s | 72^o^C/60s | 35 | 753 | (Cacciò *et al.*, 2002; Lalle *et al.*, 2005) |
|  |  | 2^o^ | **β-GIAF**  **β-GIAR** | GAACGAGATCGAGGTCCG  CTCGACGAGCTTCGTGTT | 94^o^C/45s | 55^o^C/30s | 72^o^C/60s | 35 | 511 |  |
| *Cryptosporidium* spp. | Small ribosomal subunit (18S) rRNA^a^ | 1^o^ | **SSUF1** | TTCTAGAGCTAATACATGCG | 94^o^C/45s | 55^o^C/45s | 72^o^C/60s | 35 | 1325 | (Xiao *et al.*, 1999) |
|  |  |  | **SSUR1** | CCCATTTCCTTCGAAACAGGA |  |  |  |  |  |  |
|  |  | 2^o^ | **SSUF2** | GGAAGGGTTGTATTTATTAGATAAAG | 94^o^C/45s | 55^o^C/45s | 72^o^C/60s | 35 | 826-864^b^ |  |
|  |  |  | **SSUR2** | CTCATAAGGTGCTGAAGGAGTA |  |  |  |  |  |  |
|  | 60-kDa glycoprotein (*gp60*) | 1^o^ | **GP60-Canis-F1**  **GP60-Canis-R1** | ATACTCTGGTCTCCCGTTT  GTACTCGGAAGCGGTGTA | 94^o^C/45s | 52^o^C/45s | 72^o^C/80s | 35 | 750 | (Jiang *et al.*, 2021) |
|  |  | 2^o^ | **GP60-Canis-F2**  **GP60-Canis-R2** | AAGGCGCCTCACTCATT  TCAGTTAGATATCACCCATTAA | 94^o^C/45s | 52^o^C/45s | 72^o^C/80s | 35 | 700 |  |

^a^ locus used for pathogen detection; ^b^ depending on isolate; 1^o^ – conditions for primary step of nested-PCR; 2^o^ – conditions for secondary step of nested-PCR.
